# Combination Therapy with Platelet-Rich Plasma and Epidermal Neural Crest Stem Cells Increases Treatment Efficacy in Vascular Dementia

**DOI:** 10.1155/2023/3784843

**Published:** 2023-12-18

**Authors:** Somayeh Akbari, Masoud Haghani, Mojtaba Ghobadi, Etrat Hooshmandi, Afshin Borhani Haghighi, Mohammad Saied Salehi, Sareh Pandamooz, Negar Azarpira, Afsoon Afshari, Shahrbanoo Zabihi, Marzieh Nemati, Mahnaz Bayat

**Affiliations:** ^1^Histomorphometry and Stereology Research Center, Shiraz University of Medical Sciences, Shiraz, Iran; ^2^Department of Physiology, The Medical School, Shiraz University of Medical Sciences, Shiraz, Iran; ^3^Clinical Neurology Research Centre, Shiraz University of Medical Sciences, Shiraz, Iran; ^4^Stem Cells Technology Research Center, Shiraz University of Medical Sciences, Shiraz, Iran; ^5^Shiraz Institute of Stem Cell and Regenerative Medicine, Shiraz University of Medical Sciences, Shiraz, Iran; ^6^Shiraz Nephro-Urology Research Center, Shiraz University of Medical Sciences, Shiraz, Iran

## Abstract

This study aimed to evaluate the efficacy and treatment mechanism of platelet-rich plasma (PRP) and neural crest-derived epidermal stem cells (ESCs) in their administration alone and combination in vascular dementia (VaD) model by two-vessel occlusion (2VO). *Methods*. Sixty-six rats were divided into six groups: the control, sham, 2VO + vehicle, 2VO + PRP, 2VO + ESC, and 2VO + ESC + PRP. The treated groups received 1 million cells on days 4, 14, and 21 with or without 500 *µ*l PRP (twice a week) after 2VO. The memory performance and anxiety were evaluated by behavioral tests including open field, passive avoidance, and Morris water maze. The basal-synaptic transmission (BST) and long-term potentiation (LTP) were assessed through field-potential recordings of the CA1. The mRNA expression levels of IGF-1, TGF-*β*1, PSD-95, and GSk-3*β* were measured in the rat hippocampus by quantitative reverse transcription polymerase chain reaction. *Results*. The results demonstrated impaired learning, memory, and synaptic plasticity in the 2VO rats, along with a significant decrease in the expression of IGF-1, TGF-*β*1, PSD-95, and upregulation of GSK-3*β*. Treatment with ESC alone and ESC + PRP showed similar improvements in spatial memory and LTP induction, with associated upregulation of PSD-95 and downregulation of GSK-3*β*. However, only the ESC + PRP group showed recovery in BST. Furthermore, combination therapy was more effective than PRP monotherapy for LTP and memory. *Conclusions*. The transplantation of ESC showed better effects than PRP alone, and combination therapy increased the treatment efficacy with the recovery of BST. This finding may be a clue for the combination therapy of ESC and PRP for VaD.

## 1. Introduction

In cases of aging and chronic cerebral hypoperfusion (CCH), where symptoms may not be apparent, there is a gradual increase in the risk of vascular dementia (VaD) over a period of months to years [1]. Individuals with VaD typically exhibit varying degrees of cognitive deficits [2]. In this patient, working memory loss, intellectual deficit, severe anxiety, and hallucination impaired daily activity and social relationships [3]. Unfortunately, despite the exponential increase in the incidence rate of dementia, effective treatments are still lacking. Cell therapy has shown promising results in enhancing functional recovery in irreversible neurological disorders [4]. It seems that cell therapy as a potential therapeutic approach may exert beneficial effects against senile dementia. Recently, hair follicle stem cells, which contain abundant neural stem cells, have emerged as a reliable source for cell therapy, especially in neurodegenerative disorders [5–8]. In vitro studies have reported that epidermal neural crest stem cells (EPI-NCSC) of hair follicles could differentiate into neural cells, oligodendrocytes, astrocytes, and Schwann cells [9, 10]. Our previous study found that the intravenous infusion of 1 × 10^6^ EPI-NCSC yielded better results than the 2.5 × 10^6^ cells in the two-vessel occlusion (2VO) rats [11]. Additionally, in vivo studies have shown the migration potential of EPI-NCSC to the injury side enhanced functional recovery in different animal models of neurodegenerative diseases such as ischemic stroke [8], Alzheimer's diseases (ADs) [12], peripheral nerve injury [13], and spinal cord injury [14].

Platelet-rich plasma (PRP) by releasing various types of neuroprotective growth factors has shown neuroprotective potential in experimental studies [15, 16]. In particular, PRP with cell adhesion molecules and chemotactic properties can increase the migration potential of stem cells to the site of injury [17]. Stem cell and PRP therapy were used as effective treatment methods for orthopedic injuries [18] and experimental spinal cord injuries [19].

However, the combined use of PRP and neural crest-derived epidermal stem cells (ESCs) has not yet been evaluated in VaD models. Therefore, in the present study, we aimed to investigate the combined use of 1 × 10^6^ ESC and PRP on learning–memory impairment following the 2VO model.

## 2. Materials and Methods

### 2.1. Animals and Ethics Statement

A total of 66 adult male Sprague Dawley rats, aged 6–8 weeks (bodyweight 220–250 g), were purchased from the Experimental Animal Center of Shiraz University of Medical Sciences. Animals were housed in controlled facilities: light–dark cycle (12 : 12 hr) with free access to food and water at a temperature of 23 ± 1°C and humidity of 50% ± 10%. Animal studies were conducted under the protocols and guidelines approved by the Institutional Ethics Committee of Shiraz University of Medical Sciences (IR.SUMS.REC.1399.1179).

### 2.2. Experimental Groups

After 1 week of adaptation to the new environment, the rats were randomly divided into the following groups: the control (*n* = 9), sham operation (*n* = 9), bilateral common carotid occlusion with vehicle (2VO + V (PBS); *n* = 12), and bilateral common carotid occlusion with PRP (2VO + PRP; *n* = 12); we allocated 12 rats to each transplantation group (2VO + ESC and 2VO + ESC + PRP). Unfortunately, eight rats died after 2VO surgery, and the final grouping is given below.

**Figure 1:**
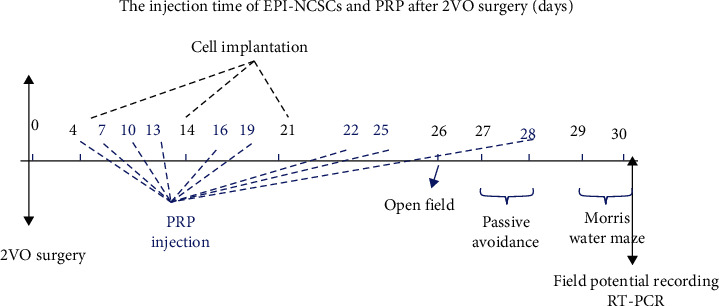
The schematic representation of different methods such as 2VO surgery, behavioral tests, and timeline in transplantation of the epidermal stem cells (ESC) and platelet-rich plasma (PRP) injection in two-vessel occlusion (2VO) rats.

The control (*n* = 9), sham operation (sham, *n* = 9), 2VO + V (*n* = 10), 2VO rats that received PRP twice-weekly from days 4 to 30 postoperatively (2VO + PRP; *n* = 9), 2VO rats that received 1 × 10^6^ cells (2VO + ESC; *n* = 11) on days 4, 14, and 21 after surgery, and 2VO rats that received combined cells and PRP (2VO + ESC + PRP; *n* = 10). In our study, there was no report of animal death following cell transplantation. Various procedures, including surgery, cell transplantation, behavioral tests, PRP injection, field potential recording, and real-time polymerase chain reaction (PCR), were performed according to the timeline, as illustrated in Figure 1.

### 2.3. Preparation of PRP

We used 30 male Sprague–Dawley rats (250–300 g) for PRP preparation. Following the cardiac puncture and whole blood extraction, the animal's blood was mixed with 3.2% sodium citrate in special tubes (Eppendorf tubes that contained sodium citrate). Then, blood samples were centrifuged for 10 min at 400×*g*. After removal of the supernatant in another centrifuge tube, it was centrifuged with higher centrifugal force at 1,000×*g* for 10 min. After discarding the upper layer, the remaining was collected as PRP and frozen at −80°C for use [20]. The percentage of the platelet was over 90% by this method and contained 1,500,000 platelets in each microliter. We used hemocytometers with Rees and Ecker method to count the platelets. The PRP was prepared according to the procedure used by Nemati et al. [21]; they also showed that the number of the platelets was 1,500,000 in each microliter. Due to the high concentration of the PRP, which may result in counting errors, it is necessary to dissolve the PRP in PBS at a one-to-one ratio to ensure an accurate platelet count. Thus, the final volume injected in each rat was 500 *µ* and contained approximately 375,000,000 platelets (250 *μ*l PRP + 250 *μ*l PBS, i.p.) [16]. The beneficial therapeutic effects of PRP injection with the same dose have been shown in our previous studies on memory impairment in animal models of vascular dementia [16] and hepatic encephalopathy [15]. The freezing and thawing method activates PRP before injection [22]. We stored PRP at −80°C maximum for 1 month [23].

### 2.4. Preparation of EPI-NCSCs

**Figure 2:**
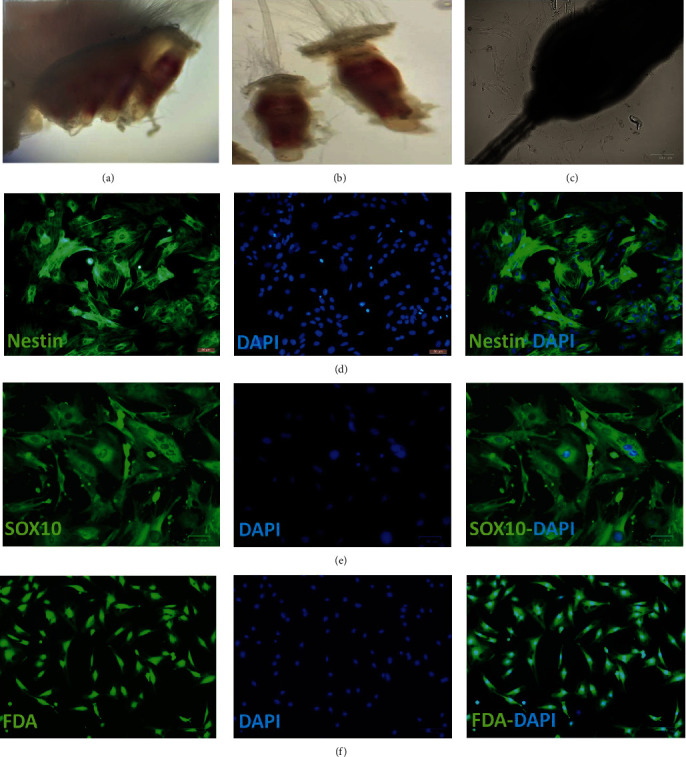
The rat whiskers pad (a). The isolated individual hair follicles (b). Seven days after explanation, the stem cells began to migrate from the hair follicle (c). Immunostaining was performed against nestin and Sox10 as neural crest stem cell markers. The actin markers with Alexa Fluor 488 conjugated secondary antibodies emitted green fluorescence, scale bar = 50 *μ*m (d, e) The viability of stem cells was also assessed by fluorescein diacetate (FDA) staining, scale bar = 100 *μ*m (f). The cell nuclei were counterstained blue with 4ʹ,6-diamidino-2-phenylindole (DAPI).

The EPI-NCSCs were obtained from individual hair follicles of the rat whiskers pad (Figure 2(a)). The isolated skin was carefully washed, and each hair follicle was longitudinally sectioned to separate the bulge area from the capsule (Figure 2(b)). The collected bulge regions were placed in a collagen-coated plate and cultured in essential medium-*α* (*α*-MEM, Sigma–Aldrich) supplemented with 10% fetal bovine serum (FBS, Gibco), 5% day-11 chick embryo extract, and 1% penicillin/streptomycin (P/S, Gibco). The culture was then incubated at 37°C in a humidified atmosphere with 5% CO_2_. Half of the culture medium was replaced daily after 7–9 days, migration of stem cells from the bulge area could be observed (Figure 2(c)); finally, these cells were detached and passaged with 0.25% trypsin/EDTA (Gibco). This procedure was described in detail in previous publications [24, 25].

### 2.5. EPI-NCSCs Verification

To verify the expanded EPI-NCSCs, immunostaining was performed using nestin and SRY-Box Transcription Factor 10 (Sox10) as neural crest stem cell markers. Briefly, the cultured EPI-NCSCs were fixed with 4% paraformaldehyde and washed with PBS containing 0.05% Tween-20. Blocking was performed using 1% bovine serum albumin containing 0.2% Triton X-100, followed by overnight incubation at 4°C with primary antibodies: mouse antinestin (1 : 50; Abcam, #ab6142) and rabbit anti-Sox-10 (1 : 100; proteintech, 10422-1-AP). The cells were incubated with goat antimouse IgG AlexaFluor488 (1 : 1,000, ThermoFisher, #A-11001) or goat antirabbit IgG AlexaFluor488 (1 : 1,000, Abcam, #ab150085) secondary antibodies at room temperature for 2 hr. Finally, the cells were counterstained with 4ʹ,6-diamidino-2-phenylindole (DAPI) (Sigma, #D9564). Images were captured with the Olympus inverted fluorescence microscope (Figure 2(d)–2(f)). EPI-NCSCs at passage number 4 were transplanted in 2VO rats. The viability of stem cells was also assessed by fluorescein diacetate (FDA) staining. In brief, EPI-NCSCs in passage number 4 were washed with PBS and fixed using 4% paraformaldehyde. Following additional washing steps, the stem cells were stained with 25 *μ*g/ml fluorescein diacetate (FDA, Sigma #F7378) and 5 *μ*g/ml Hoechst 33342 (Sigma #B2261) working solutions for 5 min. Cell nuclei were counterstained with DAPI in PBS (1 min) at room temperature in the dark. Then, the cells were washed with PBS buffer and imaged using a ZOE Fluorescent Cell Imager (Figures 2(d)–2(f)).

### 2.6. Induction of Bilateral Common Carotid Arteries Occlusion

The 2VO is one of the more practical ways and well-known tools for assessing vascular dementia. Due to the complete circle of Willis, the rats are suitable for induction of CCH by 2VO. [26]. The CCH was induced, as described previously in more detail [27]. In brief, the rats were anesthetized by intraperitoneal injection of ketamine (90 mg/kg) and xylazine (5 mg/kg). The neck area was cleansed with alcohol and povidone–iodine, and a ventral midline incision was made to expose the right and left common carotid arteries. Under a surgical microscope, the vagal nerve fibers and cervical sympathetic were carefully exposed without causing any damage to the blood vessels or nerve fibers. A small incision was made in the carotid sheath to pass a piece of 5−0 type surgical silk under the carotid artery. Right and left common carotid arteries were permanently ligated. Unlike stroke research, 2VO studies aim to investigate the long-term effects of CCH in neurodegenerative diseases [26]. However, the cerebral infarction was associated with motor impairment, but in VaD the signs and symptoms gradually worsen over time and motor impairment should not occur after 2VO [28]. To ensure that the 2VO did not lead to a stroke, all rats were evaluated for motor impairment using the NDS test. The sham operation group involved the same surgical procedure without common carotid artery ligation.

### 2.7. PRP Injection

From day 4 after the 2VO surgery until day 30, intraperitoneal injection of 500 *μ*l of PRP solution (250 *μ*l PRP + 250 *μ*l PBS) was administered twice a week. Each 500 *μ*l solution contained 375,000,000 platelets.

### 2.8. Cell Transplantation

In the 2VO + ESC and 2VO + ESC + PRP groups, 1 × 10^6^ cells in 300 *µ*l PBS were injected into the tail vein on days 4, 14, and 21 after surgery. Recently, we published an article that showed transplantation of this number of cells has the best effect on VaD [11].

Before infusion, with a pipette, the cells were gently resuspended to ensure that they were not aggregated. At the same time, animals in the 2VO + V were just injected with 300 *µ*l PBS as in the above procedures.

### 2.9. Behavioral Studies

All behavioral tests were performed between 3 pm and 6 pm. The anxiety-like behavior was evaluated using an open-field test on day 26 after 2VO. The passive avoidance test was performed on days 27 and 28. By Morris water maze (MWM) tests, the spatial memory performance was evaluated on days 29 and 30 post-2VO surgery. An experimenter blindly tested the animals. One of the personnel managed the cell implantation, and another completed a behavioral assessment, ensuring that the person who performed the behavioral testing was blind to the type of animal groups. Data were collected by a video image motion analyzer (Ethovision, Noldus Information Technology, Netherlands).

#### 2.9.1. Open-Field Test

The locomotion and anxiety-like behaviors were assessed using an open-field apparatus, which consists of a square Plexiglas box (90 (W) × 90 (L) with a height of 45 cm). The field floor was divided into 16 squares and defined as central and peripheral regions. The animals were observed for 15 min, and the video tracking software recorded the time spent in the peripheral and central regions (in seconds), and the number of grooming of the animal was recorded and analyzed off-line, using EthoVision (Noldus Information Technology, Netherlands) [29].

#### 2.9.2. Passive Avoidance Test

A shuttle box was used for the evaluation of fear learning and memory according to our previous published studies [16, 30]. The shuttle box consisted of two chambers, one white and one black, separated by a sliding guillotine door made of Plexiglas. On the 27th day after surgery, the fear learning was achieved with punishment by an electrical foot shock (0.5 mA, 50 Hz, 2 s once) upon entrance to the black chamber. This learning trial was repeated every 5 min until the rats no longer crossed from the white to the black side. The number of electrical shocks was considered a learning index for each animal. The step-through latency (STL) is the time each animal stays on the white side before crossing through the gate to the black chamber. On the 28th day of postsurgery, the STL time was recorded as fear memory index without any electric shock.

#### 2.9.3. Morris Water Maze

The MWM test was used to evaluate spatial learning and memory, as previously described in more detail [29]. Briefly, memory acquisition was performed on the 29th day of postsurgery, consisting of 12 trials divided into three blocks. During each trial, one rat was allowed to swim in a water maze for 60 s, and the time spent in the target quadrant to find the hidden platform is considered as a measure of spatial learning. The intervals between trials and blocks were set at 35 s and 30 min, respectively. On the 30th day of postsurgery, during the probe trial, the platform was removed, and the percentage of the time spent in the target quadrant was recorded as indexes for spatial memory retention. A longer duration spent in the target quadrant indicates better spatial memory retention.

### 2.10. Field Potential Recording

On the 30th day of postsurgery, the field potential recording was performed from the hippocampal CA1 area, as described previously in more detail [16, 31]. Briefly, the head of the anesthetized rats was fixed in a stereotaxic apparatus. The Schaffer collateral pathway (-4AP and 3 L) and the CA1 region (-3AP and 2 L) were drilled for the insertion of stimulating and recording electrodes (0.2 mm diameter, Advent, UK). The typical shape of the field excitatory post-synaptic potential (fEPSP) wave is considered as an index for confirmation of the correct position of the electrode in the CA1 area. After a 15-min rest, the input/output curve was plotted using increment stimulation intensity from 50 to 1,200 *μ*A. In baseline recording and high-frequency stimulation (HFS), we used 40% and 80% of the maximum amplitude responses recorded from the input–output curve. The baseline fEPSP was recorded for 25 min before HFS. For evaluation of the short-term plasticity, the PPR (pulse2/pulse1) was calculated at inter-stimulus intervals (ISIs) of 25, 50, 100, 150, 200, and 250 ms before and after HFS. The HFS consisted of three trains (0.1 Hz), each train being composed of 20 pulses at a frequency of 200 Hz. After HFS, recording continued for 60 min at the intensity of stimulation at 40% of the maximum response in the I/O curve. The percentage of change of the fEPSP amplitude after HFS to the baseline value is considered an index for long-term potentiation (LTP) induction.

### 2.11. Quantitative Reverse Transcription PCR

The total RNA was extracted from 50 mg of rat hippocampi using RNAX Plus (Sinaclon, Tehran, Iran) and then diluted to a concentration of 500 ng/*µ*l with RNase-free water. The purity of total RNA was assessed with the NanoDrop 2000/2000c spectrophotometer (Thermo Scientific, USA). Reverse transcription of the RNA into cDNA was performed using an Easy cDNA synthesis kit (Parstous, Mashhad, Iran). qPCR was performed using a RealQ Plus 2x Master Mix Green (Ampliqon, Denmark) in a reaction volume of 10 *µ*l with a StepOnePlus real-time PCR system (Thermo Fisher Scientific) using the following thermocycling conditions: 95°C for 600 s, followed by 40 cycles of primer specific temperature for 5 s and 72°C for 60 s and 95°C for 15 s, primer specific temperature for 60 s and 95°C for 15 s. We used the primer sequences as shown in Table 1. All values were normalized to the housekeeping gene GAPDH. Finally, *ΔΔ*Ct method was applied to compare the relative gene expression. We technically repeated the sample three times.

### 2.12. Statistical Analysis

The means of values in passive avoidance, open field, water maze, PPR, EPSP slope in the I/O curve, and relative mRNA expression levels in six groups with normal distribution were compared using one-way ANOVA with Tukey's post hoc test. All values are expressed as mean ± SEM. The normalized amplitude of EPSP before and after HFS was compared with the paired *t*-test. Two-way repeated-measures ANOVA was employed for evaluation of the fEPSP changes in repeated times after delivery of HFS and time spent in three blocks of the MWM test. All the data analyses were performed using PRISM 6 software, and the significance level was set at *P* < 0.05.

## 3. Results

### 3.1. Behavioral Tests

#### 3.1.1. Open-Field Test

**Figure 3:**
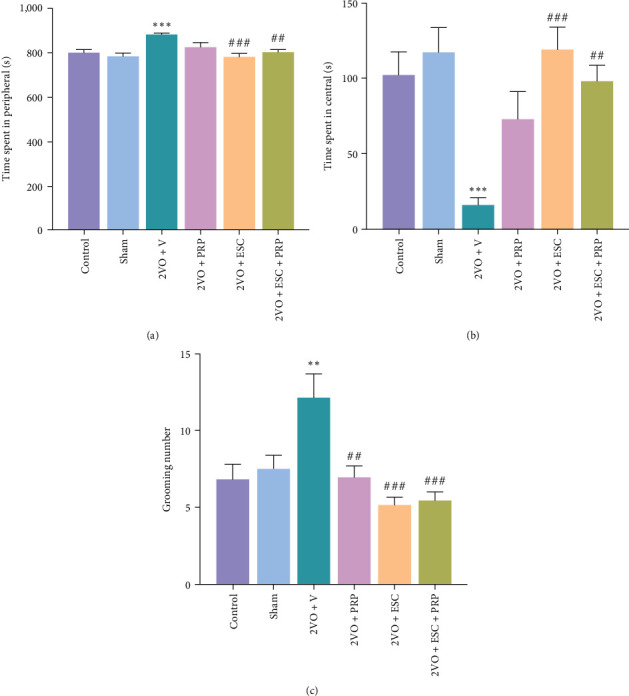
The evaluation of anxiety-like behaviors in the studied groups by open-field tasks. Transplantation of epidermal stem cells (ESC) in 2VO + ESC and combined treatment in 2VO + ESC + PRP groups showed a significant decrease in the peripheral time and an increase in the central time relative to the values in the 2VO + V group (a, b). In PRP, ESC, and combined groups, the grooming number significantly decreased compared to the 2VO + V group (c). The values are shown as mean ± SEM. Significant differences with respect to the sham ( ^*∗∗*^*P* < 0.01,  ^*∗∗∗*^*P* < 0.001) and 2VO + V ( ^*##*^*P* < 0.01, ^###^*P* < 0.001). Control (*n* = 9), sham (*n* = 9), 2VO + V (*n* = 10), 2VO + PRP (*n* = 9), 2VO + ESC (*n* = 11), and 2VO + ESC + PRP (*n* = 10). One-way ANOVA with the post hoc test.

In the 2VO + V group, there was a significant increase in the time spent in the peripheral area compared to the sham groups (884.8 ± 4.4 s vs. 782.2 ± 15.91 s; *P* < 0.001), while the time spent in the center decreased (16.18 ± 4.6 s vs. 117.8 ± 15.81 s; *P* < 0.001) (Figures 3(a) and 3(b)). However, cell transplantation alone in the 2VO + ESC group improved both the time spent in the peripheral (780.9 ± 15.33 s) and central (119.l ± 15.3 s) regions compared to the 2VO + V group (both; *P* < 0.001). Similarly, combination therapy also recovered the time spent in the peripheral (801.8 ± 10.81 s) and central (98.15 ± 10.7 s) regions, which were significant different from the 2VO + V group (both; *P* < 0.01). Furthermore, PRP treatment in the 2VO + PRP group failed to improve the time spent in the peripheral and central regions compared to the 2VO + V group (Figures 3(a) and 3(b)).

In the 2VO + V group, the grooming number increased relative to the sham group (12.20 ± 1.511 vs. 7.55 ± 0.86; *P* < 0.01), while both combination and single therapy improved the grooming number to the same level. The treatment with PRP, cell transplantation alone, and combination therapy significantly ((*F*(5, 52) = 8.059; *P* < 0.0001) decreased the grooming number to 7.0 ± 0.7, 5.15 ± 0.52 and 5.5 ± 0.54, respectively, compared to the 2VO + V group (*P* < 0.01 and *P* < 0.001) (Figure 3(c)).

#### 3.1.2. Passive Avoidance Test

**Figure 4:**
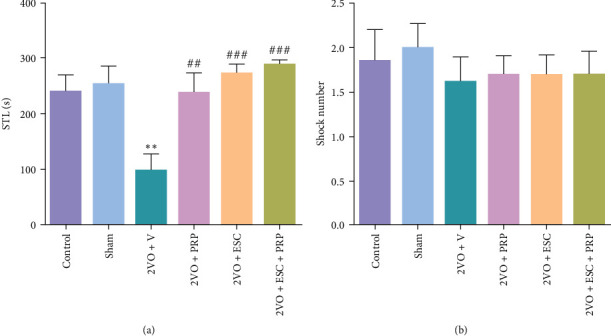
The evaluation of fear memory in the studied groups by passive avoidance task. Significantly higher STL times relative to the 2VO + V group were found in the 2VO + ESC, 2VO + PRP and in the 2VO + ESC + PRP groups (a). The number of shocks was at the same level in all groups (b). The values are shown as mean ± SEM. Significant differences with respect to the sham ( ^*∗∗*^*P* < 0.01) and 2VO + V (^##^*P* < 0.01, ^###^*P* < 0.001). Control (*n* = 9), sham (*n* = 9), 2VO + V (*n* = 10), 2VO + PRP (*n* = 9), 2VO + ESC (*n* = 11), and 2VO + ESC + PRP (*n* = 10). One-way ANOVA with the post hoc test.

The duration spent by each animal in the light chamber before entering the dark chamber, known as the STL time, was measured as an index for fear memory. A decrease in STL time indicates impairment in fear memory. As depicted in Figure 4(a), the STL time in the 2VO + V group was decreased compared to the sham group (98.5 ± 29.4 vs. 256.7 ± 29.7; *P* < 0.01). We found that PRP injection significantly increased the STL in the 2VO + PRP group (240.3 ± 34.6 s) compared to the 2VO + V group (*P* < 0.01). Significantly higher STL time for entrance to the dark chamber relative to the 2VO + V group were 273.2 ± 16.9 s and 292.7 ± 5.2 (*P* < 0.001) (*F*(5, 52) = 7.845, *P*=0.0001), respectively. There was no significant difference in shock numbers between all the studied groups (Figure 4(b)).

#### 3.1.3. Morris Water Maze Test

**Figure 5:**
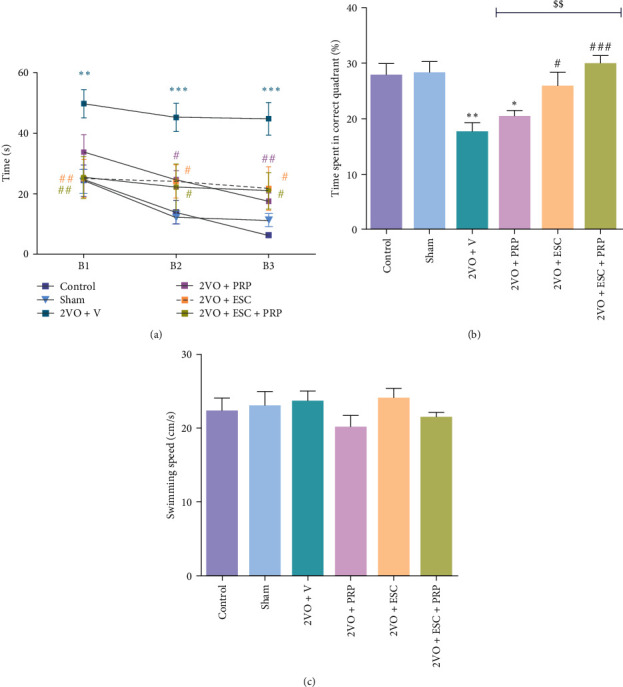
The evaluation of spatial learning and memory by Morris water maze in all groups. The PRP injection in the 2VO + PRP group led to a significant decline in the escape latency time in the third block compared to the 2VO + V group. The escape latency time during the three blocks in the 2VO + ESC and 2VO + ESC + PRP groups significantly decreased compared to the 2VO + V group (two-way ANOVA) (a). In the probe trial, better memory retention was recorded in the 2VO + ESC and 2VO + ESC + PRP groups relative to the 2VO + V group (b). (One-way ANOVA with post hoc test). Swimming speed was the same in all the studied groups (c). Significant differences with respect to the sham ( ^*∗*^*P* < 0.05,  ^*∗∗*^*P* < 0.01, and  ^*∗∗∗*^*P* < 0.001) and 2VO + V (^#^*P* < 0.05, ^##^*P* < 0.01, ^###^*P* < 0.001). Significant differences between 2VO + ESC + PRP group respect to the 2VO + PRP group (^$$^*P* < 0.01). The values are shown as mean ± SEM. Control (*n* = 9), sham (*n* = 9), 2VO + V (*n* = 10), 2VO + PRP (*n* = 9), 2VO + ESC (*n* = 11), and 2VO + ESC + PRP (*n* = 10).

In this test, the time spent for finding the hidden platform, known as the escape latency time, was measured during 12 trials in three blocks. As shown in Figure 5(a), in the 2VO + V rats, escape latency was significantly higher compared to the sham group in the first (49.6 ± 4.6 vs. 24 ± 3.9; *P* < 0.01), second (45.1 ± 4.6 s vs. 12.1 ± 2.2; *P* < 0.001), and third blocks (44.7 ± 5.3 s vs. 11. 2 ± 2.2; *P* < 0.001). The PRP infusion in the 2VO + PRP group led to a significant decline in the escape latency time in the second (24.5 ± 2.9 s) and third block (17.5 ± 2.6 s) compared to the 2VO + V group (*P* < 0.05 and *P* < 0.01). We found a significant decline in the escape latency time during the three blocks in the 2VO + ESC and 2VO + ESC + PRP groups compared to the 2VO + V group (block 1; *P* < 0.01, blocks 2 and 3; *P* < 0.05).

Twenty-four hours after the last learning trial, spatial memory retention was evaluated. After removing the platform, the percentage of swimming time spent in the target quadrant for each rat was calculated as an index for memory retention (Figure 5(b)). A decrease in this index indicates memory impairment. This percentage significantly decreased in the 2VO + V group compared to the sham group (17.8% ± 1.3% vs. 28.4% ± 1.9%; *P* < 0.01). We found better memory retention in the 2VO + ESC (25.9% ± 2.3%) and 2VO + ESC + PRP (30.1% ± 1.3%) groups relative to the 2VO + V group (*P* < 0.05 and *P* < 0.001) (*F*(5, 52) = 7.21, *P* < 0.0001). Additionally, in the 2VO + PRP group (20.5% ± 0.95%), injection of PRP alone did not improve memory retention relative to 2VO + V. Notably, the combined use of PRP and ESC resulted in a significant increase in the time spent in the target quadrant compared to the PRP group (30.1% ± 1.3% vs. 20.5% ± 0.95%; *P* < 0.01). There was no significant difference in the swimming speed between all groups (Figure 5(c)).

### 3.2. Field Potential Recording at Schaffer Collateral-CA1 Synapse in 2VO Rats

#### 3.2.1. Basal Synaptic Transmission

**Figure 6:**
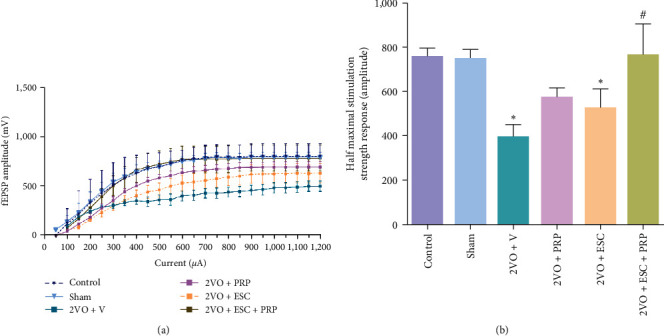
The basal synaptic transmission (BST) of the hippocampal CA1 neurons was assessed by input/output curve in all groups. The input/output curve (a). A significant right and downward shift in the input/output curve of 2VO rats shows BST decline following the 2VO model. A significant decrease in the half-maximal fEPSP amplitude in the 2VO + V group relative to the sham group (b). The combination therapy of PRP and cells showed better effects and a significant increase in the half-maximum response compared to 2VO + V group. Significant differences were relative to the sham ( ^*∗*^*P* < 0.05) and 2VO + V (^#^*P* < 0.05). Control (*n* = 8), sham (*n* = 7), 2VO + V (*n* = 8), 2VO + PRP (*n* = 8), 2VO + ESC (*n* = 8), and 2VO + ESC + PRP (*n* = 7). One-way ANOVA with the post hoc test.

Figure 6(a) demonstrates a functional decline of basal-synaptic transmission (BST) in the 2VO + V group compared to the sham group by a downward and right shift in the I/O curve. One-way ANOVA analysis showed a significant reduction in the half-maximal fEPSP amplitude in the 2VO + V group to the sham group (398.7 ± 50.2 vs. 749.4 ± 38.5; *P* < 0.05). Although the cell therapy alone failed to recover the half-maximal fEPSP amplitude, PRP improved this parameter to some extent. In addition, the combination therapy of PRP and cells showed better effects and significantly increased the half-maximum response compared to the 2VO + V group (765.8 ± 137; *P* < 0.01) (*F*(5, 40) = 4.663, *P*=0.0019) (Figure 6(b)).

#### 3.2.2. Short-Term Synaptic Plasticity

**Figure 7:**
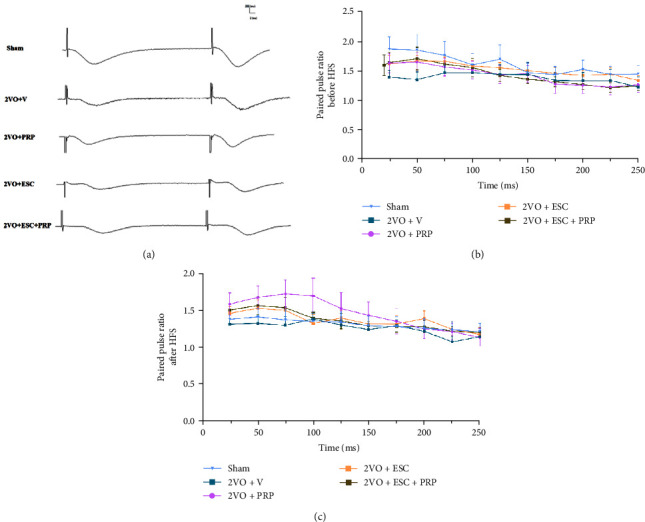
The evaluation of short-term synaptic plasticity in studied groups by paired-puls ratio (PPR) calculation. For different ISIs (25−250 ms). The sample traces of responses (a), the PPR was plotted by linear graph before (b), and after (c) HFS delivery. There was no significant difference between all the studied groups before or after HFS in the PPR. The values are shown as mean ± SEM. Control (*n* = 8), sham (*n* = 7), 2VO + V (*n* = 8), 2VO + PRP (*n* = 8), 2VO + ESC (*n* = 8), and 2VO + ESC + PRP (*n* = 7).

Figure 7(a)–7(c) illustrates the evaluation of the short-term plasticity by calculating the paired-pulse ratio (PPR), which represents the ratio of fEPSP amplitude of the second response to the first response at ISIs 25–250 ms. A PPR value greater than 1 indicates facilitation in synaptic transmission. There was no significant difference in PPR between all the groups (Figure 7(a)–7(c)).

#### 3.2.3. Long-Term Potentiation (LTP)

**Figure 8:**
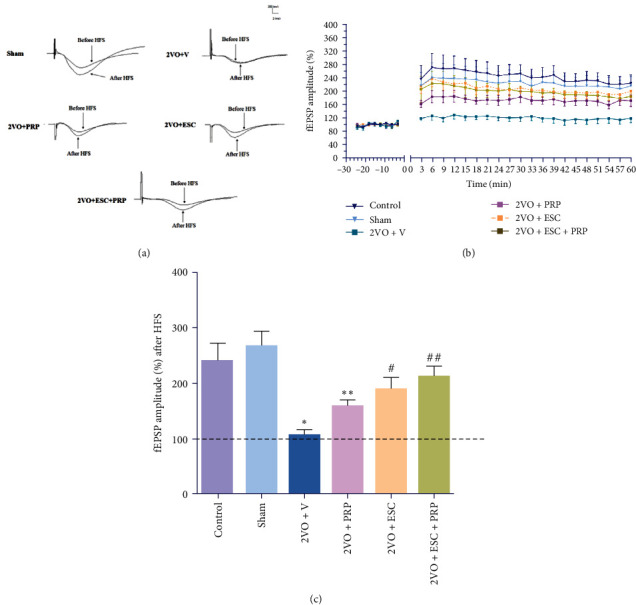
The comparison of long-term potentiation (LTP) induction between different studied groups. LTP induction in sample traces of responses (a). After high-frequency stimulation (HFS), the percentage of the change of the fEPSP amplitude compared to the baseline was compared between all groups (b). The means of fEPSP amplitude after HFS were compared between all groups (c). Significant differences with respect to the sham ( ^*∗*^*P* < 0.05,  ^*∗∗*^*P* < 0.01) and 2VO + V (^#^*P* < 0.05, ^##^*P* < 0.01). The values are shown as mean ± SEM. Control (*n* = 8), sham (*n* = 7), 2VO + V (*n* = 8), 2VO + PRP (*n* = 8), 2VO + ESC (*n* = 8), and 2VO + ESC + PRP (*n* = 7).

We evaluated the hippocampal LTP in all groups for 60 min after HFS. In different groups, we assessed the sample traces of responses (Figure 8(a)) and the mean fEPSP magnitude (Figure 8(b)). In the 2VO + V group, the magnitude of LTP was lower compared to the sham group (111.1% ± 5.9% vs. 268.1% ± 26.1%; *P* < 0.001) (Figure 8(a)–8(c)). Although the PRP treatment in the 2VO + PRP group still showed a significant decrease relative to the sham group (*P* < 0.01), we found a significant LTP induction in the 2VO + ESC (190.3% ± 19.9%; *P* < 0.05) and 2VO + ESC + PRP groups (214.2% ± 17.3%; *P* < 0.01) relative to the 2VO + V group (*F*(5, 40) = 8.588; *P* < 0.0001) as much as the sham group (Figure 8(c)).

### 3.3. mRNA Expression Levels of IGF1, TGF-*β*1, PSD-95, and GSk-3*β* in the Rat Hippocampus

**Figure 9:**
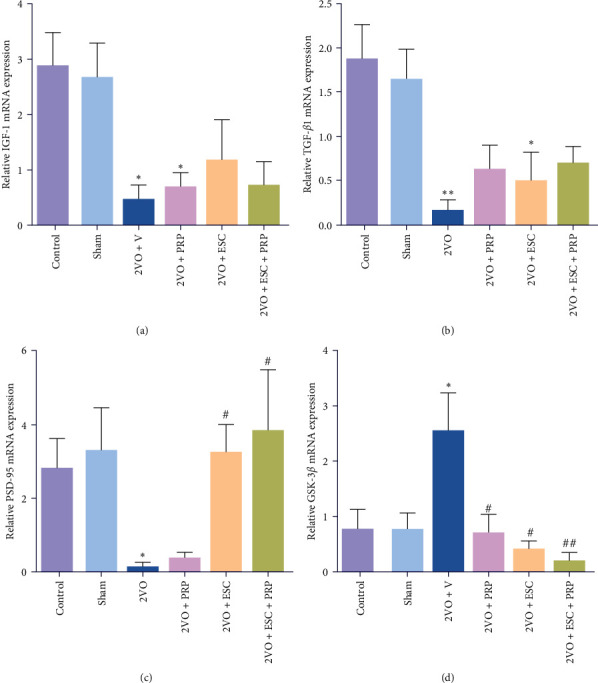
mRNA expression levels of IGF1, TGF-*β*1, PSD-95, and GSk-3*β* in the rat hippocampus (a–d). The values are shown as mean ± SEM. Significant differences with respect to the sham ( ^*∗*^*P* < 0.05,  ^*∗∗*^*P* < 0.01) and 2VO + V (^#^*P* < 0.05, ^##^*P* < 0.01). The values are shown as mean ± SEM. Control (*n* = 6), sham (*n* = 6), 2VO + V (*n* = 5), 2VO + PRP (*n* = 5), 2VO + ESC (*n* = 5), and 2VO + ESC + PRP (*n* = 5).

The hippocampal mRNA expression level of IGF-1, PSD-95, and TGF-*β*1 was significantly decreased in the 2VO + V group compared with the sham group (*P* < 0.05 and *P* < 0.01, respectively). Conversely, the mRNA expression levels of GSK-3*β* significantly increased in the 2VO + V group (2.55 ± 0.68) compared to the sham group (0.77 ± 0.29), *P* < 0.05 (Figure 9(a)–9(d)). However, the GSK-3*β* expression decreased following treatment only with PRP (0.70 ± 0.32), ESC (0.41 ± 0.13; *P* < 0.05), and combined treatment (0.20 ± 0.14; *P* < 0.01) compared to the 2VO + V (Figure 9(d)). Also, significant increases were seen in PSD-95 expression following cell transplantation (3.27 ± 0.72) and combination therapy (3.85 ± 1.65) relative to the 2VO + V group (0.17 ± 0.09; *P* < 0.05) (Figure 9(c)), but treatment only with PRP failed to recover the PSD-95 level. The same profile was seen for IGF-1; cell transplantation and combination therapy partially recovered the IGF-1 expression, but treatment with only PRP did not show any effects on IGF-1. Moreover, we found that, following treatment with PRP and combination therapy, the TGF- *β*1 expression was partially recovered, but treatment with only ESC could not show any effects on TGF-*β*1 expression in the 2VO rats (Figure 9(b)).

## 4. Discussion

In the present study, we aimed to investigate the advantage of the combined use of ESC and PRP relative to ESC transplantation or PRP injection when used alone to recover memory and synaptic plasticity impairment in 2VO rats.

The allograft transplantation of 1 million ESC without immunosuppression was repeated three times after 2VO surgery. In our previous study, the transplantation of 1 million cells showed better functional recovery in the 2VO rats to 2.5 million cell grafting [11].

Our data indicated impairment in fear memory, spatial learning memory, LTP, BST, and anxiety-like behavior in the 2VO rats compared to the sham group. Moreover, in the 2VO model, the mRNA expression levels of IGF-1, PSD-95, and TGF-*β*1 significantly decreased, and GSK-3*β* expression significantly increased relative to the sham group. In addition, following treatment with PRP alone, we found significant recovery in fear memory, spatial learning, and grooming number relative to the 2VO rats. Also, PRP injections twice weekly could not recover BST and LTP impairment after 2VO. However, in the 2VO + PRP group, the expression of TGF-*β*1 showed a partial increase, and GSK-3*β* expression significantly decreased relative to the 2VO rats. Particularly, stem cell therapy showed significant performance recovery in all behavioral tests and LTP induction relative to the 2VO model. The results of RT-PCR showed a significant increase in PSD-95 and a significant decrease in GSK-3*β* expression in 2VO + ESC compared to 2VO + V. Furthermore, similar to the 2VO + ESC group in the combined treatment group, we found significant recovery in fear memory, spatial learning memory, grooming number, LTP, and BST relative to 2VO rats, but combination therapy showed better outcomes and results for BST compared to ESC alone. Therefore, we found BST recovery only in the combined treatment group, while LTP was induced in both ESC and ESC + PRP groups. A previous study reported that for the rescue of BST, a longer period may be needed relative to LTP [32]. It seems that the combined use of PRP with ESC could accelerate BST recovery relative to PRP or ESC when used alone. Thus, after combination therapy, BST recovery might be a key player in LTP induction. We also had the best recovery in spatial memory in the combined group, which was accompanied by BST recovery. Several reports have indicated that spatial memory loss occurs in the early stage of AD, and synaptic dysfunction rather than neuronal death can represent the primary cause of spatial memory impairment [33, 34].

At individual synapses, BST involves the neurotransmitters released in response to a single action potential [35]. In the field potential recording, the slope of the input/output curve following increasing stimulus intensity from 100 to 1,200 *µ*A shows the strength of BST or synaptic excitability. Several factors affecting the decline of BST after cerebral hypoperfusion include enhancing inhibitory synaptic transmission, reducing evoked transmitter release through elevated presynaptic calcium [36], increasing the number of silent hippocampal synapses by 30%–55% [36], dendritic degeneration, neuronal death [37], and impairing the presynaptic plasticity impairment [38]. The possible mechanisms for BST recovery after cell therapy might be forming new synapses between the host cells and grafted cells and/or increasing the number of functional synapses through neurotrophic support. The spontaneous excitatory postsynaptic currents in graft-derived cells were recorded seven weeks after transplantation [39]. However, another study has recorded the postsynaptic currents in grafted cells at least 4 weeks posttransplantation [40, 41]. Our study found BST improvement only after combination therapy in the 2VO + ESC + PRP group.

Unfortunately, we did not use the stem cell tracking method. Previous studies demonstrated the homing potential and fate of EPI-NCSC to the sites of the inflammatory area following brain injury [8, 12, 42]. In our study, based on the time elapsed between transplantation and field potential recording (4 weeks), we can make this hypothesis that better functional performance in behavioral test and synaptic plasticity following stem cell therapy might be mediated primarily by synaptotrophic support of the host cells and then new synaptic circuit between the host and grafted cells. It seems that the coadministration of PRP with ESC can promote the new bidirectional synapse between the host and transplanted cells to achieve more effective synaptic transmission than ESC or PRP alone. Perhaps the synergistic effect of combination therapy in releasing the growth factors on the host neural circuits caused this functional improvement without any new circuits between the host and grafted cells. Previous reports have suggested the hippocampal synaptic plasticity improvement following stem cell transplantation in 2VO and myocardial infarction models [11, 43]. It has been indicated that EPI-NCSCs could express the vascular endothelial growth factor (VEGF), neurotrophic growth factor (NGF), and brain-derived neurotrophic factor (BDNF), [24, 44, 45]. BDNF can enhance synaptic transmission [46] and synaptogenesis [47] and improve BST, LTP [32], and synaptic interactions [48]. It also can increase axonal branching by activating TrkB and TrkC [49, 50]. These results showed that ESC-derived growth factors might effectively improve the synaptic transmission.

We also found that the hippocampal expression of TGF-*β*1 was significantly decreased 4 weeks after 2VO. Sun et al. [51] reported a significant increase in the TGF-*β*1 concentration in the corpus callosum of 2VO rats on days 3 and 7 after the surgery model; however, on day 28, it decreased and reached the same levels as the sham group. We also found a significant downregulation of TGF-*β*1 in the 2VO + ESC group compared to the sham group and a partially nonsignificant increase in TGF-*β*1 following treatment with PRP alone and combination method. Therefore, it is possible that PRP injection had a key role in hippocampal TGF-*β*1 expression 4 weeks after 2VO in the 2VO + PRP and 2VO + ESC + PRP groups. Several studies reported the TGF-*β*1 upregulation following treatment with PRP in different types of tissue injuries [52, 53]. Moreover, TGF-*β*1 is one of the several PRP-derived growth factors that can stimulate the proliferation and differentiation of different stem cells in injury models [54, 55]. The administration of TGF-*β*1 could recover memory loss and hippocampal synaptic plasticity through the PI3K/Akt signaling pathway in an Alzheimer's model [56]. It was reported that synaptic plasticity and the number and length of the dendritic spine are facilitated by PRP-derived growth factors [57, 58]. These might help to justify LTP recovery in the 2VO + PRP and combined treatment groups. In addition, we found the GSK-3*β* upregulation in the hippocampus of 2VO rats and GSK-3*β* downregulation in all types of treatment, including ESC, PRP (*P* < 0.05), and ESC + PRP (*P* < 0.01). Previous studies have indicated the overactivation of GSK-3*β* in the hippocampus of 2VO rats, while its suppression has shown the therapeutic target for 2VO memory deficit [59]. An increase in the activity of GSK-3*β* in AD brains increases tau phosphorylation and contributes to the formation of neurofibrillary tangles and amyloid plaques [60]. GSK-3*β* activity blocks the synaptic LTP and induces long-term depression [61].

We also found a significant downregulation of PSD-95 in 2VO rats. At the same time, the mRNA expression of PSD-95 as a synaptic plasticity-associated protein significantly increased following injection of ESC and ESC + PRP without a significant difference between the two groups. Zhu et al. [62] reported that the level of PSD-95 was significantly increased in a transgenic mouse model of AD following neural stem cell transplantation. It seems that stem cell-derived growth factors facilitate the mRNA expression of PSD-95 in the hippocampus of animals.

In our study, an open-field test showed a significant increase in anxiety-like behavior of the 2VO rats compared to the sham group. Cell transplantation in the 2VO + ESC group was associated with reduced anxiety behavior based on the increase in the central time and a decrease in peripheral time and grooming number compared with the 2VO + V rats. We found a significant decrease in the amount of grooming behavior in the PRP groups without any change in the central and peripheral time. Previous studies showed that the grooming number alone could be interpreted as decision-making [63]. A decrease in grooming numbers without any recovery in the central and peripheral time may not be a reliable indicator of animal stress and anxiety [64, 65]. People with dementia have difficulties performing a variety of decision-making in daily living [66]. Brain regions associated with decision-making are frontal, temporal, and parietal regions, and these areas are vulnerable to age-related change [67].

Experimental research showed that the MWM is reliable for evaluating hippocampal function (place learning). However, in the passive avoidance disturbance, damaged regions extend from the hippocampus to the amygdala and surrounding area [68]. Following PRP injection, we could see a significant recovery in decision-making and fear memory without any improvement in spatial memory. Based on our results, there is the possibility that the neurotropic effect of PRP-derived growth factors in a large area of the brain might contribute to decision-making and fear-memory improvement. We also found that the 2VO + PRP + ESC and 2VO + ESC groups recovered from spatial memory disturbance in 2VO rats. Thus, it seems that spatial memory recovery needs the neurotropic effects of ESC-derived growth factors and/or new circuits between the host cells and grafted cells or an increase in hippocampal neurogenesis. Several trophic factors can influence hippocampal neurogenesis including BDNF [69], insulin-like growth factor-I (IGF-I) [70], NGF [71], VEGF, and fibroblast growth factor-2 (FGF-2). EPI-NCSCs-derived growth factors include BDNF, NGF, and VEGF [24, 44, 45].

The multipotential cells of the hair follicles are an accessible stem cell source. Autotransplantation of these cells can be a promising therapeutic approach in neurodegenerative disorders. However, further studies are required before use in a clinical trial to clarify the migration, homing potential, and differentiation of EPI-NCSC when used with PRP compared to EPI-NCSC alone.

## 5. Conclusion

Our results indicated that the transplantation of EPI-NCSC showed better performance in spatial memory improvement than PRP alone, and combined use of EPI-NCSC with PRP could restore BST in 2VO rats. This finding may be a clue for the combination therapy of EPI-NCSC and PRP for vascular dementia.

## Figures and Tables

**Table 1:** Primer sequences (5′–3′) used in qPCR.

Gene name (ID)	Forward primer (F1) (5′–3′) Reverse primer (R1) (5′–3′)	Ta(c)	Product length (BP)	Exons
IGF1 (insulin growth factor) Gene ID: 24482	F1-TGGTGGACGCTCTTCAGTTC R1-TCCGGAAGCAACACTCATCC	57–61	123	2–3
Gsk-3*β* (glycogen synthase kinase-3 beta) Gene ID: 84027	F1-AGCTGATCTTTGGAGCCACC R1-CTGATCCACACCACTGTCCC	56–61	119	6–7
TGF-*β*1 (transforming growth factor, beta 1) Gene ID: 59086	F1-TGACATGAACCGACCCTTCC R1-TGCCGTACACAGCAGTTCTT	57–62	138	5–6
PSD95 (discs large MAGUK scaffold protein 4) Gene ID: 29495	F1-CTGCATCCTTGCGAAGCAAC R1-AAGAAACCGCAGTCCTTGGT	57–62	85	11–12

## Data Availability

Data supporting this research article are available on request.
